# Artificial intelligence: is it a friend or foe of physicians?

**DOI:** 10.31744/einstein_journal/2019ED4982

**Published:** 2019-04-25

**Affiliations:** 1 Global Health and Tropical Medicine, Instituto de Higiene e Medicina Tropical, Universidade Nova de Lisboa, Lisboa, Portugal.

Health systems need to be strengthening to face new challenges. Demographic growth must increase the demand for more services, and resulting comorbidities are making systems more expensive. New technologies can contribute positively to transform our society, but, in the healthcare area, this transformation is still late. There is a disproportion between launching innovative services and such services economic impact. Two ideas have dominated the field according to experts, that is, the skeptical picture about the benefit of health technologies and the need to reorganize health because of low level of economic growth, and increasing demand for services. However, others are more convinced of the promise of these technologies innovation for healthcare, especially related with the economic gains that digital technologies will bring. Digital technologies are already producing huge benefits to health information. Emerging evidences have already show some beneficial effects of digital services, despite the many challenges that remain linked to their implementation in health context.^(^ ^1^ ^,^ ^2^ ^)^

Technology is a crucial factor, but only when well-aligned to the care process. Artificial intelligence (AI), as well as Big Data, and Internet-of-Things, are extremely relevant, but there is a need of evidence or, even, a guarantee that these technologies are useful. The massive use of data combined with AI can, perhaps, from 5 to 10 years, allow better comprehension of health systems dynamic (*e.g.,* user's behavior, use of services, epidemics, etc.), better use of resources, and improve the creation of knowledge. IBM, Google and other companies have invested large amount of money in AI, but, so far, available AI resources are not ready to be applied in large scale in the healthcare area.^(^ ^3^ ^)^

The key for good application of AI would be the existence of qualified physicians.^(^ ^4^ ^)^ A qualified physician would know how to deal with care teams using AI to improve quality. Artificial intelligence must develop itself within the next years, and physicians have an important role of integrating AI in their daily practice in order to guarantee that AI will help, and not complicate processes. Initially, we will probably have systems that do not work well.^(^ ^3^ ^)^ If physicians wish, the AI can be aligned to their decision making. If physician do not take part of AI development, global enterprises would impose AI models in health that can produce less benefits, and to exert more control over physicians.

New technologies offer important opportunities for new digital services that will allow a more active role of users in health management. Physicians need more information and to use AI to find out essential standards to take care of patients. The AI is also a challenge of collaboration between health institutions and research centers. We should take advantage of the excitement within scientific community and develop a system that show benefits of AI for physicians’ practice and to the healthcare system.^(^ ^1^ ^,^ ^5^ ^)^

For this reason, acquisition of AI skills by physician is fundamental. The implementation of services based on AI requires adjustments in services delivery and organization of medical consultation. The sustainability of health care depends on how efficient is the design of the new health digital services, and how AI will help improve physician's performance in the future.^(^ ^3^ ^,^ ^5^ ^,^ ^6^ ^)^
